# Immunogenicity of two COVID-19 vaccines used in India: An observational cohort study in health care workers from a tertiary care hospital

**DOI:** 10.3389/fimmu.2022.928501

**Published:** 2022-09-23

**Authors:** Vidya Arankalle, Archana Kulkarni-Munje, Ruta Kulkarni, Sonali Palkar, Rahul Patil, Jitendra Oswal, Sanjay Lalwani, Akhilesh Chandra Mishra

**Affiliations:** ^1^ Department of Communicable Diseases, Interactive Research School for Health Affairs, Bharati Vidyapeeth (Deemed to be) University, Pune, Maharashtra, India; ^2^ Department of Pediatrics, Bharati Vidyapeeth Medical College, Bharati Vidyapeeth (Deemed to be) University, Pune, Maharashtra, India; ^3^ Bharati Vidyapeeth Medical College, Bharati Vidyapeeth (Deemed to be) University, Pune, Maharashtra, India

**Keywords:** SARS CoV-2, COVISHIELD, COVAXIN, immunogenicity, T cell response

## Abstract

COVID-19 pandemic witnessed rapid development and use of several vaccines. In India, a country-wide immunization was initiated in January 2021. COVISHIELD, the chimpanzee adenoviral-vectored vaccine with full-length SARS-COV-2 spike insert and COVAXIN, the whole virus-inactivated vaccines were used. To assess and compare immune response of health-care-workers to COVISHIELD (n=187) and COVAXIN (n=21), blood samples were collected pre-vaccination, 1month post-1/post-2 doses and 6months post-dose-2 and tested for IgG-anti-SARS-CoV-2 (ELISA) and neutralizing (Nab,PRNT50) antibodies. Spike-protein-specific T cells were quantitated by IFN-γ-ELISPOT. In pre-vaccination-antibody-negative COVISHIELD recipients (pre-negatives, n=120), %Nab seroconversion (median, IQR Nab titers) increased from 55.1% (16, 2.5-36.3) post-dose-1 to 95.6% (64.5, 4.5-154.2, p<0.001) post-dose-2 that were independent of age/gender/BMI. Nab response was higher among pre-positives with hybrid immunity at all-time points (p<0.01-0.0001) and independent of age/gender/BMI/Comorbidities. Post-dose-2-seroconversion (50%, p<0.001) and Nab titers (6.75, 2.5-24.8, p<0.001) in COVAXIN-recipients were lower than COVISHIELD. COVAXIN elicited a superior IFN-γ-T cell response as measured by ELISPOT (100%; 1226, 811-1532 spot forming units, SFU/million PBMCs v/s 57.8%; 21.7,1.6-169.2; p<0.001). At 6months, 28.3% (15/53) COVISHIELD and 3/3COVAXIN recipients were Nab-negative. T cell response remained unchanged. During immunization, COVID-19 cases were detected among COVISHIELD (n=4) and COVAXIN (n=2) recipients. At 6months, 9cases were recorded in COVISHIELD-recipients. This first-time, systematic, real-world assessment and long-term follow up revealed generation of higher neutralizing antibody titers by COVISHIELD and stronger T-cell response by COVAXIN. Diminished Nab titers at 6months emphasize early booster. Immunogenicity/efficacy of vaccines will change with the progression of the pandemic needing careful evaluations in the field-settings.

## Introduction

The current COVID-19 pandemic caused by SARS-CoV-2 continues to affect global population. In India, after initial COVID-19 cases among travelers from endemic countries, local transmission was established in different states at different times. Maharashtra state and Pune city wherein this study was conducted were the hotspots. Highest number of cases during the first and second waves in India (Pune) were 97,859 (2,120) in Sept 2020 and 414,433 (7,010) in May 2021 (1–3).

COVID-19 pandemic witnessed rapid development and extensive use of vaccines employing conventional and novel platforms. In India, “Emergency Use Authorization” was granted to two vaccines, COVISHIELD and COVAXIN, both produced in India. COVISHIELD is the trade name for ChAdOx1 nCoV-19 recombinant vaccine developed by the University of Oxford (Oxford, UK). This vaccine was first manufactured by AstraZeneca, UK as AZD1222 (the coded research name) and Vaxzevria (the trade name) and subsequently by Serum Institute of India Pvt. Ltd. (SIIPL). COVISHIELD is fully equivalent to the Vaxzevria vaccine. COVAXIN is the trade name for the whole-virion inactivated, Algel-imidazoquinoline (IMDG) adjuvanted SARS-CoV-2 vaccine (BBV152) developed by Bharat Biotech International Ltd, (BBIL), India, in collaboration with the Indian Council of Medical Research and, manufactured at BBIL. IMDG is a TLR7/8 agonist used to augment cell-mediated responses. In addition to spike-protein, the target protein of COVISHIELD, the other envelope proteins (nucleoprotein, membrane and envelop) are also present.

The government of India initiated a nationwide immunization program on 16th January 2021. Health care workers (HCW) was the obvious first priority group followed by frontline workers and senior citizens. As on 28th February 2022, over 1870 million vaccine doses have been administered (COVISHIELD, 79.3% and COVAXIN, 20.7%). The results of phase-I (4), phase-II (5) and phase-III (6) clinical trials of COVAXIN have been reported. However, though the vaccine manufactured at AstraZeneca has been studied extensively (7–10), similar data for COVISHIELD and Indian population are not yet available in the public domain. For the assessment of true benefit of a vaccine to any population, it is essential to assess the immune responses in field setting. This is especially true for COVID-19 as the vaccines were developed and approved globally for human use in record time. For a large and diverse country like India and considering the scale of the immunization drive in the shortest possible time, it would be essential to assess immune response of recipients of both the vaccines (and the newer vaccines when introduced) in the field setting. We report immunogenicity of two vaccines in HCW vaccinated at a single center during the early phase of the national program and serologic follow up for 6months.

## Materials and methods

Recruitment, vaccination, and sampling were done at Bharati Vidyapeeth (deemed to be) University Medical College and hospital (BVDTUMCH), a tertiary care hospital and designated immunization center for COVID vaccines, at Pune, India. The study was approved by the “Human Ethics Committee’’ of BVDUMCH (No: BVDUMC/IEC/185A). Written informed consent was obtained from all the participants. The vaccines were provided by the government and immunization was dependent on the availability of a particular vaccine on a given day and not as per choice.

### Vaccines and vaccination schedules

#### Vaccines

1. COVISHIELD: One vaccine dose contains 5X10^5^ viral particles.

2. COVAXIN: One dose contains 6ug of whole-virion inactivated SARS CoV-2 antigen.

#### Vaccination schedules

Vaccine supply was made by the government through local public health administration. Vaccines were administered irrespective of prior COVID-19. Depending on the type of the vaccine supplied on a day, inoculations were performed by trained staff. Eligibility for vaccination was strictly followed as per the recommendations of the manufacturers. At the time of conducting this study, the national policy was to immunize HCW with two doses of both the vaccines, 4weeks apart.

### Study population and sample collection

The number of study participants was solely dependent on the feasibility of immunological analyses and enrolment at a single center during first 3weeks of the study initiation. In December 2020, IgG-anti-SARS-CoV-2 positivity among blood donors from BVDTUMCH was found to be 39.3% (our unpublished observations) indicative of exposure of a large proportion of the population to SARS-CoV-2. Considering feasibility of immunologic assays, 40% positivity and 50% dropouts at the time of sampling at 1month post-2^nd^dose, a sample size of 400/vaccine was estimated. This would allow us follow up of 120 vaccinees negative (pre-negatives) and 80 vaccinees positive (pre-positives) for IgG-anti-SARS-CoV-2 antibodies before vaccination for each of the two vaccines. In view of the small numbers of COVAXIN recipients during first 3weeks, enrolment was continued for additional 2weeks. History of (H/O) COVID was obtained before each sampling. For the subjects included in the final analysis, H/O comorbidities was obtained telephonically. During the routine health check-up conducted ~two months before vaccination, Body-Mass-Index (BMI) was available for the majority; fresh measurements were taken for the others. Blood samples were collected before vaccination, before 2^nd^-dose and, 1month/6months post-dose-2, in EDTA tubes. PBMCs were separated within 4hours of blood collection by ficoll‐histopaque based density gradient method. PBMCs and plasma samples were stored at -80^0^C in aliquots.

### Serology

All the samples were tested for the [1] presence of IgG-anti-SARS-CoV-2 antibodies by ELISA (anti-spike antibodies, SCoV-2 Detect IgG ELISA, InBios International, Inc., USA). and [2] presence/titers of neutralizing antibodies (Nabs) by 50% plaque reduction neutralization test (PRNT_50_) using live virus, as described earlier (11). SARS-CoV-2 strain (8004/IND/2020/Pune) used for PRNT belongs to Clade G (Genbank accession No: MT416726). Based on testing of plasma samples from 61 blood donors collected during 2017-19, the cut off value for a positive test was a titer of ≥10 (11). For statistical analysis, negatives were assigned a titer value of 2.5.

### IFN-γ ELISPOT

For the assessment of T cell response, SARS CoV-2-spike-protein-specific ELISPOT was done using Human IFN-γ ELISPOT^Pro^ kit (Mabtech) according to the manufacturer’s instructions. Briefly, cryopreserved PBMCs were revived and rested overnight at 37°C in a humidified CO2 incubator. After trypan blue based live cell count, approximately, 4 × 10^5^ PBMCs/well were stimulated for 24hrs in duplicate with peptide pool comprising 15mer overlapping-peptides spanning the whole Spike-protein (Source-BEI NIH, USA) at 2 μg ml−1 concentration of individual peptides. Negative controls comprising 0.1%DMSO, complete culture media and positive control [purified anti-CD3, (Mabtech) & CD28 (BioLegend) stimulation] were included for each sample. Spots were counted using CTL S6 Macroanalyser (CTL Biospot) and presented as spot forming units (SFU)/million PBMCs. Based on the testing of 22 SARS CoV-2 IgG-negative subjects, the cut off value (mean+3 SD) was ≥14 SFU/million PBMCs. **Supplementary Figure 2** depicts representative image of ELISPOT plate scan.

### Statistical analysis

Graph pad Prism (5.01 version) and “R” software (version 4.05) were used for analyses. For comparison between the groups, Mann Whitney U test and z tests were performed for comparisons between the groups and two proportions respectively. For the paired comparisons of PRNT_50_ titers and T cell responses, Wilcoxon Signed Rank sum test was used. Simple linear regression was performed to understand linear relationship between different covariates and antibody titers. Except for 9 subjects for whom BMI data was not available, all the other information was available for all the participants.

## Results

### COVISHIELD

#### Enrolment, vaccination, and demographics

The details of the participants at different time points are depicted in **Supplementary Figure 1**. At pre-vaccination, blood samples were collected from 425 willing participants. Evidence of previous exposure to SARS-CoV-2 as indicated by IgG-anti-SARS-CoV-2 positivity was seen in 163 (38.3%) while H/O COVID-19 as confirmed by viral RNA positivity was given by 57 participants (13.4%). Thus, the ratio clinical: subclinical infections was 1:1.86. Nab titers in vaccinees with clinical disease (median=57, Interquartile Range, IQR=22-97) did not differ from those having subclinical infection (median=84, IQR=35-147) (p=0.44). Among symptomatic individuals, disease severity was mild (n=52), moderate (n=3) and severe (n=2). Subsequently, only 222/425 initial participants were ready for the further follow up. Of these, sampling after second dose was possible for 187.

Characteristics of the pre-negative and pre-positive vaccinees sampled after two vaccine doses are summarized in **Table 1**. Except for age group, the other parameters were comparable. Only 4 vaccinees were > 65years age and all were IgG-anti-SARS-CoV-2 negative prior to vaccination. Prior antibody positivity was higher among vaccinees aged ¾ 55years whereas the proportion of pre-negatives was higher in >55years age group (p=0.005 for both). Among comorbidities, diabetes mellitus, hypertension and cardiovascular disease were present in the majority (31/34).

**Table 1:** Demographic characteristics of the COVISHIELD recipients analyzed post-2^nd^ dose.

Parameters	Total	IgG Negatives	IgG positives	p value
**Number**	187	120 (64.1%)	67 (35.8%)	<0.001
**Males**	88 (47%)	53 (44.1)	35 (52.2%)	0.36
**Females**	99 (52.9%)	67 (55.8%)	32 (47.7%)	0.36
**Age ¾ 55 years (Median-35.5)**	159 (85%)	95 (79.1%)	64 (95.5%)	0.005
**Age >55 years (Median-62.8)**	28 (14.9%)	25 (20.8%)	3 (4.4%)	0.005
**Comorbidities**	34/182 (18.6%)	24/120 (20%)	10/62 (16.1%)	0.66
**High BMI (≥25)**	80*/182	48**/107 (43.9%)	32***/62(43.3%)	0.4

Obese (BMI>30): *21, **10, ***11.

#### Breakthrough infections during the study period

Before the post-2^nd^-dose sampling, 4 mild COVID-19 cases were recorded, none in the elderly. All were pre-negatives. The disease onset was post-1^st^ dose in one and post-2^nd^-dose in three. These were removed from further analysis. Between 2^nd^-dose and sampling at 6months, 9vaccinees developed mild clinical disease. Except one (60years male), others were in 27-54 years age group. None of pre-positives reported clinical COVID-19 during the follow up.

#### Post-immunization antibody responses

As vaccines were administered irrespective of prior antibody positivity, both pre-negatives and pre-positives received the vaccine. These groups were analyzed separately.

### Immune response to COVISHIELD

#### Dynamics of antibodies among pre-negatives

Post-dose-1, 95.6% vaccinees developed binding antibodies (ELISA), only 55.1% being neutralizing. Post-2^nd^-dose, 95% elicited neutralizing antibodies, 100% ELISA reactive. The ages of Nab negatives (6/120) were 22F, 26F, 36F, 63F, 41M and 60M. **Supplementary Table 1** presents Nab titre-wise response 1month post complete immunization. The PRNT titers were <100 in 65/120 (54.2%) and ≥ 100 in 49/120 (40.8%) vaccinees. **Supplementary Table 2** displays distribution of PRNT titers.

**Figure 1:**
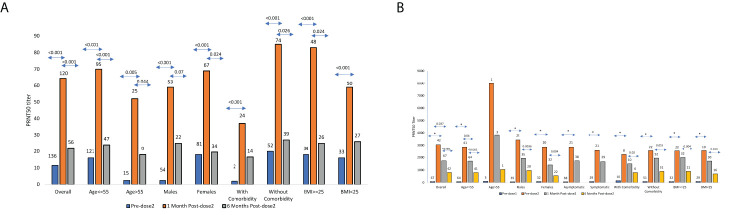
**(A)** SARS CoV-2 PRNT50 titers in Pre-negatives PRNT50 titers (median ± SE) among COVISHIELD vaccine recipients (n = 120) negative for IgG-anti-SARS-CoV-2 antibodies prior to vaccination and post 1month/6months post-2^nd^ dose. The numbers above the bars represent the number of samples examined in different categories. **(B)** SARS CoV-2 PRNT50 titers in Pre-positivesPRNT50 titers (median ± SE) among COVISHIELD vaccine recipients (n = 67) positive for IgG-anti-SARS-CoV-2 antibodies prior to vaccination and predose-2, post 1month/6months of 2^nd^ dose. The numbers above the bars represent the number of samples examined in different categories * Indicates p value < 0.001.

Among pre-negatives (**Figure 1A**), the median Nab titer increased 4fold from 16 at post-first-dose (IQR 2.5-36.3) to 64.5 (IQR 34.5-154.2) after 2^nd^-dose (p<0.001). Similar increase was seen in both the age groups (p<0.001) and gender (p<0.001), participants with normal (<0.001) or elevated BMI (p<0.001) and with or without comorbidity (<0.001 for both). Nab titers after both the doses were independent of age (p=0.25), gender (p=0.90) and BMI (p=0.43). Vaccinees without comorbidities mounted higher Nab titers than those with such conditions, both after 1^st^ (p=0.003) and second (p=0.004) dose. Comorbidities were present in 0.18% of the younger and 0.44% of the older age groups (p=0.027).

At 6months post-2^nd^-dose, 56 vaccinees were available (**Figure 1A**; **Supplementary Table 2**). Three non-responders continued to be Nab and ELISA negative. Of note, 15/53 (28.3%) vaccinees turned Nab negative, all positive in ELISA. Overall, the titers declined from median 64.5 (IQR 34.5-154.2) to median 22 (IQR=2.5-87, p<0.001; **Supplementary Table 2**). Interestingly, the decline was highly significant in both ¾ 55 and > 55 years age groups (p<0.001 and 0.044 respectively), females (p=0.024), vaccinees without comorbidities (p=0.026) or higher BMI (p=0.024).

#### Dynamics of antibodies among pre-positives

##### At 1month post-complete immunization with two doses

Post-1^st^ dose, the median Nab titer increased 40.7fold from 75 (IQR 29-129) before vaccination to 3050 (1282-3998, p< 0.001). However, post-2^nd^-dose, no boosting effect was seen (**Figure 1B**). In fact, the median titers declined 1.75fold to 1740 (911-3116, p=0.0376). Age-wise comparison was not possible as only 3 vaccinees were >55years age. The rise in Nab titers was comparable when other variables were considered (p<0.001 for all). Though decline in antibody titers was seen in all the groups, the difference was significant only in ≤55 years age group (p=0.04). For the rest, the difference was insignificant (p=0.082 for females and 0.12-0.5 for other variables). Nab titers post-both doses were independent of Age (p=0.25), gender (p=0.102), BMI (p=0.48), and comorbidities (p=0.62) (**Figure 1B**). As reflected by simple regression analysis, none of these variables were associated with PRNT titers post-dose-1 (p=0.46-0.78). For post-dose-2, Nab levels post-dose-1 was the only independent variable influencing antibody response (p<0.001, Pearsons’s correlation coefficient r=0.84).

##### At 6months post-2^nd^-dose

All the 42 vaccinees sampled at 6months were positive for neutralizing antibodies (**Figure 1B**; **Supplementary Table 2**). Of these, 41 were ¾55 years age and circulated significantly lower Nab titers (median 803, IQR 348-1278) when compared to post-2^nd^-dose (median 1740, IQR 911-3116, p<0.001) (**Figure 1B**; **Supplementary Table 2**). Further, the decline was significant irrespective of the gender (p=0.0036 for males and 0.004 for females), BMI (< 25, p=0.016, >25, p=0.0004) and comorbidity (with: p=0.02 and without: p=0.003). Of note, the number of vaccinees with comorbidities was small at both the time points (10 after complete immunization and 6 at 6months).

#### T cell response at 1 and 6months post-2^nd^ dose

**Figure 2:**
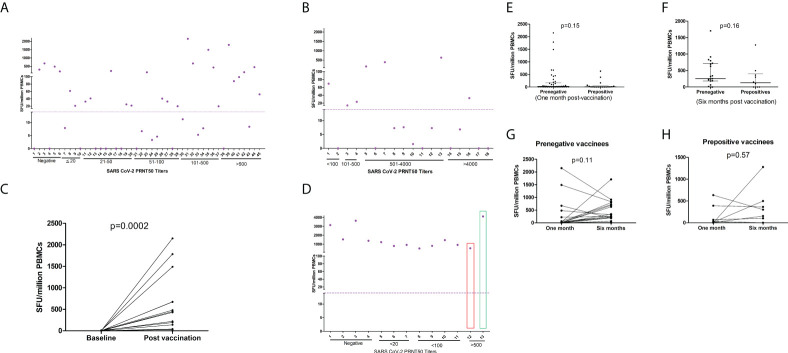
T cell responses to COVISHIELD and COVAXIN. Spike protein peptide-specific T cell responses among **(A)** pre-vaccination IgG-anti-SARS-CoV-2 negative COVISHIELD recipients (n = 45) and **(B)** pre-vaccination IgG-anti-SARS-CoV2 positive COVISHIELD recipients (n = 18). **(C)** IFN-γ ELISpot responses in 16 pre-IgG negative COVISHIELD recipients prior to post-2nd dose vaccination. **(D)** Pre-vaccination IgG-anti-SARS-CoV-2 negative COVAXIN recipients (n = 12) and one IgG-anti-SARS-CoV-2 positive. One IgG negative recipient (Number 13, green rectangle) developed clinical COVID-19 post 2nd dose; sampled 3weeks post-diagnosis. Before vaccination, the pre-positive vaccinee (Number 12) had PRNT titer of 119 that increased to 469 post-second dose). Purple circles (IFN-γ ELISpot, Spot forming Units/million PBMCs), depict corresponding values for the individual patients. Dotted line show cut off values for IFN-γ ELISpot. ELISpot=Enzyme-Linked Immunospot; PBMC=peripheral blood mononuclear cells. Comparison of T cell response (IFN-γ ELISPOT) (Mann Whitney U test) between prepositives and prenegatives **(E)** at one month of postvaccination **(F)** at six months of postvaccination Comparison of T cell responses (Wilcoxon signed rank sum test) at one month and six months post vaccination **(G)** Prenegative vaccinees **(H)** Prepositive vaccinees.

For understanding antigen-specific T cell response at 1month post-immunization, γ-interferon ELISPOT was performed in 63 COVISHIELD recipients (**Supplementary Table 3**; **Figures 2A, B**). The selection of the subjects was based on post-dose-2 PRNT titers. Among the pre-negatives (n=45), 57.8% were responsive to IFN-γ ELISPOT. The positivity rate for IFN-γ response among pre-positives (n=18) was 61.1%. Of the 6 PRNT-non-responders, 4 displayed T cell response. Testing of 16 paired PBMC samples from pre-negatives revealed absence of any reactivity prior to vaccination and robust, variable IFN-γ ELISpot response post-vaccination (**Figure 2C**). T cell response was independent of PRNT titers (r=0.02).

At 6months post-2^nd^-dose, samples could be obtained from 31 participants (**Figures 2E, F**). In both pre-negatives (n=21, **Figure 2G**) and prepositives (n=10, **Figure 2H**), IFN- γ spot forming units at both time points were comparable (p=0.11 and 0.57 respectively).

#### Comparison among vaccinees without or with prior exposure to SARS-CoV-2

Post-dose-1, titers among the pre-positives were 190.6fold higher than the pre-negatives. However, due to the lack of boosting effect post-dose-2 in the pre-positives and four-fold rise in pre-negatives, the difference was reduced to 27.2fold. At both time points, Nab titers among pre-positives were higher than the pre-negatives (p<0.001) and not influenced by age, gender, BMI and comorbidities (p<0.001 for all).

At 6months, none (pre-positives) and 28.3% (pre-negatives) vaccinees were Nab negative. Nab titers continued to be lower in the pre-negatives (median 22, IQR=2.5-87) than the pre-positives (median 810, IQR=371-1297, p<0.001). Similar trend was seen in all the categories. Surprisingly, pre-negative vaccinees with comorbidities (n=39) or BMI≥25 (n=26) did not exhibit significant decline in Nab titers (p=0.11 and 0.16 respectively). Among prepositives, significant decline was seen in these groups (n=6 and 21; p=0.02 and 0.0004 respectively).

IFN-γ-specific T cell responses were not different among pre-negatives and pre-positives at both the time points (**Figures 2E, F**). The median SFU/million PBMCs among pre-negatives/pre-positives at 1month post-2^nd^-dose were 21.7 (IQR-1.6-169.2)/7.8 (IQR=0-41.9 p=0.15) and 256 (IQR=182-721/128 (IQR=0-396, p=0.16) at 6months (**Figures 2G, H**).

### Immune response to COVAXIN

#### At 1month post-complete immunization with two doses

During the stipulated period, we could recruit only 65 individuals. However, 44 of these received second dose at different centers and hence blood samples could not be collected. The data is limited to only 21 COVAXIN recipients. Eight were ¾ 55 years (23-50years; 4males and 4females) and 13 were > 55 years old (57-81years; 10males and 3females); 2/8 and 1/13 respectively were IgG-SARS-CoV-2 positive before vaccination. Two of the pre-negatives (65M and 81M) developed clinical COVID-19 post-2^nd^-dose; the 65M diagnosed on the 12th day post-2^nd^-dose had mild disease and 3weeks later the Nab titer was 3123. The 81M was ELISA/PRNT negative after 1^st^ dose, developed COVID-19 four weeks post-2^nd^-dose. He was hospitalized and recovered without oxygen support. No sample was collected. Of the remaining 16 pre-negatives, %seroconversion post-second dose was 62.5% (ELISA) and 50% (PRNT, 4/8 each in ¾ and > 55years; median titer 6.75, IQR 2.5-24.75). Nab titers among three pre-antibody positives increased from median 119 (IQR 112.5-318.5) to 469 (median 680, IQR 574.5-1049). Due to small numbers, further analysis was not possible.

**Figure 2D** depicts T cell response elicited by COVAXIN recipients (n=13). IFN-γ response detected by ELISPOT was elicited by all the participants (100%). Among 11 pre-negatives, the median SFU was 1226 (522-3628). Importantly, IFN-γ responses among vaccinees with no/low neutralizing antibodies were similar to the pre-positive vaccinee (31M, PRNT titer469) and the one developing mild disease post-second dose (65M, PRNT titer 3123).

#### At 6months post-2^nd^-dose

At this point, samples from 3/11 pre-negative COVAXIN recipients could be obtained. All were negative in PRNT as well as ELISA. At 1month post-dose-2, one of the vaccinees was ELISA positive/Nab negative and for the remaining two Nab titers were <25. None of these subjects reported history of clinical COVID-19. T cell responses as determined by IFN-γ ELISPOT were comparable; SFU/million cells were 919, 1380 and 522 (at 1month post-2^nd^-dose) and 754, 1085 and 625 (at 6months) respectively.

#### Comparison of immune response to COVISHIELD and COVAXIN vaccines

In view of the lower response against COVAXIN and the small numbers available, we compared characteristics of both the vaccinees (**Table 2**). In the pre-negatives, Nab positivity and titers were lower in the COVAXIN group (p<0.001), but, comparable in vaccinees >55years (p=0.89 and 0.25 respectively). Among pre-positives, the titers post-dose-2 were higher with COVISHIELD (n=67, median titer 1740) than COVAXIN (n=3, median titer 680). Despite higher age, COVAXIN induced superior T cell response as measured by ELISPOT (56.5fold, p<0.001).

**Table 2:** Comparison of study participants and immune response of pre-antibody negative COVISHIELD or COVAXIN vaccine recipients at 1month post-second dose.

Parameters	COVISHIELD	COVAXIN	P value
Total number analysed post-2^nd^ dose	187	21	<0.001
Age > 55 years	28 (14.9%)	13 (61.9%)	<0.001
Pre-vaccination positives	67 (35.8%)	3 (14.2%)	0.047
**At post-2^nd^ dose among pre-negatives**			
IgG positives/No tested (%) (ELISA)	120/120 (100)	10/16 (62.5)	<0.001
Nab positives/No tested (%) Nab titers: Median (IQR)	114/120 (95) 64.5 (34.5-154.2)	8/16 (50) 6.75(2.5-4.75)	<0.001 <0.001
Nab positives/No tested in Age>55 years Nab titers: Median (IQR)	23/25 52 (26-154)	7/10 37.5 (22-83)	0.25 0.89
Nab positives/No tested in Age<55 years Nab titers: Median (IQR)	91/95 70 (36-149)	2/6 19.5 (18-21)	<0.001 0.045
Number of ELISPOT Positives (%) Median SFU/million PBMC (IQR)	26/45 (57.8) 21.7 (1.6-169.2)	11/11 (100) 1226 (811-1532)	0.008 <0.0001

## Discussion

We provide a detailed analysis of immunogenicity of COVISHIELD (Nab antibodies by PRNT and T cell response), the Indian version of AstraZeneca vaccine in a field setting from India and follow up for 6months post-vaccination. Since this vaccine is administered to ~80% of the Indian population (1), the information is of significance. The study was initiated as soon as the Indian government announced vaccination plan with HCW as the priority group. Though our aim was to compare immune response of Indian subjects to both the vaccines used, we were not able to follow up required number of COVAXIN recipients leading to very limited data for the vaccine. This remains a major limitation of our study. Nonetheless, certain indicators were identified. Here, it may be emphasized that the vaccine efficacy was tested against a highly transmissible Delta variant in a natural setting.

Our study revealed that COVISHIELD-vaccine was immunogenic and elicited Nabs in 95% of the infection-naïve recipients. When Nab response of AstraZeneca vaccine administered at similar doses and interval is considered (9), post-1^st^-dose response of COVISHIELD seems inferior (seroconversion 62.5%/100%; median Nab titers 16/218). This difference may be related pre-vaccination Nab status; all pre-negatives, this study) and 23 (AstraZeneca). For post-2^nd^-dose samples, PRNT was not used for AstraZeneca vaccine. Nab titers recorded in pre-positives (median 64.5) are in line with an earlier report of lower Nab titers for the adenovirus-vector-based-vaccines than for mRNA-vaccines (AZD1222, 11.9-19.9fold and Ad26.COV2.S, 15.3-25.6fold) (12). Recently, COVISHIELD was found to be effective in reducing SARS-CoV-2 infections and severity in a highly transmissible setting from India (13).

Irrespective of the platform used, time-dependent waning of antibodies has been observed for all the COVID vaccines studied so far (14–16). In line with these observations, we noticed significant decline in Nab titers. Among infection-naïve vaccinees, COVISHIELD-induced Nab titers at 6months were 2.9fold (median 22) lower than post-2^nd^ dose. Of the 120 pre-negatives, 3 breakthrough infections with mild disease were recorded within 1month post-2^nd^-dose. Of concern, 28.3% (15/53) vaccinees turned Nab negative at 6months while 5.4% (3/53) developed mild breakthrough infections. Thus, almost 1/3^rd^ vaccinees were susceptible at 6months. The observed better protection with 3doses than 2doses in Israel is noteworthy (17). The government of India has introduced a booster at 9months that needs to be reduced to 6months or earlier.

Lower Nab titers coupled with diminished neutralization potential against variants of concern (VOCs) (18–20) have led to administration of 3doses or boosters (21–24) leading to better antibody titers against VOCs. Boosters with heterologous vaccines have been tried with promising results (25–27) and may be a better strategy.

In the context of hybrid immunity, while confirming reports with other vaccines (28–30), Nab titers among pre-positives were higher than pre-negatives after 1^st^ (190fold), 2^nd^ (27.2fold) dose and at 6months (36.8fold) indicating possible protection from the emerging variants. This group did not report any breakthrough infection or antibody negativity. Though one dose is enough for generating high tittered Nab antibodies, lack of facilities for pre-vaccination screening with an efficient rapid test for a large country like India, wastage of one dose for naturally infected individuals seems unavoidable. In April 2021, we recorded 60% IgG-anti-SARS-CoV-2 positivity among blood donors without history of COVID when the second wave was at its peak at Pune (unpublished observations). Though blood donors do not represent the general population, it may be surmised that with such a high infection rate, vaccination should be able to generate long term protection, even from the diversified variants. A retrospective cohort study from Sweden recorded one-does and 2-dose hybrid immunity to be associated with a lower risk of COVID-19 hospitalization than natural immunity (31). Only vaccinated individuals were not considered.

Lack of sufficient number of COVAXIN recipients did affect our study wherein remarkably lower antibody response was noted. Of note, COVAXIN group included higher proportion of >55 years aged participants that are likely to induce lower antibody response (**Table 2**). Here, two studies employing large number of both the vaccinees from India are noteworthy. Anti-spike-ab positivity and titers (ELISA) were higher with COVISHIELD (97.8%, 115.5AU/ml; n=370); than COVAXIN (79.3%, 51AU/ml; n=87) (32). A 6months follow up of >300 vaccinees each receiving COVISHIELD or COVAXIN displayed 2-fold and 4-fold decrease in anti-spike-IgG titers (16). Of the 81 breakthrough infections, 63% were in COVAXIN recipients. Taken together, COVAXIN seems to produce lower humoral response.

T cell immunity is an important contributor to recovery in SARS-CoV-2 infection (33). We could demonstrate T cell responses in the majority (77.8%) of COVISHIELD-recipients (**Figure 2A, B**). Importantly, 4/6 non-responders elicited spike-specific T cell response. Despite small numbers and higher age, COVXIN-recipients elicited 100% and stronger T cell response that can be attributed to IMDG adjuvant known to augment cellular immunity. Our observations of diminished humoral and unchanged T cell response at 6months are similar to BNT162b2 (14, 34). In view of the possible role of T cell responses in protection despite lower neutralizing antibodies (35), whether COVAXIN recipients with robust Th1-driven T cell response can be protected in the absence of/lower neutralizing antibodies needs to be evaluated. Very recently, COVAXIN was shown to induce robust immune memory to SARS-CoV-2 and alpha/beta/delta variants persisting for at least 6 months post-vaccination (36).

Humoral immune response is affected by several confounding factors such as of age, gender, comorbidity and elevated BMI. Comorbidity was identified as the only variable associated with lower Nab response post-first and second dose (p=0.003 and 0.004). Association of comorbidities with vaccine-induced immune response in elderly is documented (37). Lack of influence of age on antibody response could be because only 4 individuals >65 years age. Though females are better antibody producers to vaccines, we did not find any difference. A study from Korea observed higher titers (p<0.05) among females immunized with AstraZeneca vaccine (38). This could have been due to inclusion of 74% females. Among pre-positives (**Figure 1B**), Nab response was independent of prior symptomatic, predominantly mild or asymptomatic infections. This is in accordance with our earlier observations of similar titers in patients with mild or asymptomatic infection (11). Chronic hepatitis B and H/O tuberculosis were associated with risk of infection among residents of congregate residential facilities from a northern state (13).

In conclusion, though the number of COVAXIN recipients is small, our data show that immune responses of Indian population to COVISHIELD and COVAXIN are characterized respectively by stronger humoral and cellular responses. A sharp decline in Nab titers/positivity at 6months post-COVISHIELD vaccination emphasize need for additional dose(s)/early booster. Vaccinees with hybrid immunity continue to circulate higher Nab titers even at 6months confirming broader immune response and protection from breakthrough clinical infections. With the increasing numbers of infections in both vaccinated and unvaccinated individuals, majority of the population should develop high antibody titers that can protect from further reinfections from related variants and progression to severe disease needing hospitalizations. However, with the possibility of emergence of distinctly different variants, development of vaccines eliciting broader and higher immune response remains the key in COVID control.

## Data availability statement

The raw data supporting the conclusions of this article will be made available by the authors, without undue reservation.

## Ethics statement

The studies involving human participants were reviewed and approved by Institutional Ethics Committee of Bharati Vidyapeeth (Deemed to be University) Medical College, Pune. The patients/participants provided their written informed consent to participate in this study.

## Author contributions

VA conceived, designed the study, and wrote the manuscript. AK-M & RK were responsible for ELISPOT and PRNT work respectively. SP, JO, and SL were responsible for the recruitment of vaccine recipients and collection of samples/relevant information. RP was responsible for statistical analysis. AM reviewed the results and commented on the manuscript. All authors contributed to the article and approved the submitted version.

## Funding

This work was partially supported by a grant from Department of Biotechnology- Biotechnology Industry Research Assistance Council (DBT-BIRAC), India, under the “ Innovate in India(i3)” program of National Biopharma mission (grant No- BIRAC/BT/NBM0095/02/18).

## Acknowledgments

The authors thank National Biopharma Mission, BIRAC, Department of Biotechnology, and Government of India for partial funding of the project. The authors thank Ms Shraddha Hattangadi, Mr Aniket Amlekar, Ms. Sawani Karandikar, Ms Prajakta Rane, Ms Muskan Thakur, Ms Priya Wadhwani, Ms Meghana Walke and Mr Tushar Bhosale for excellent technical assistance. The staff of BVDTUMCH involved in vaccination and blood sample collection is thankfully acknowledged. The following reagent was obtained through BEI Resources, NIAID, NIH: Peptide Array, SARS-Related Coronavirus 2 Spike (S) Glycoprotein, NR-52402.

## Conflict of interest

The authors declare that the research was conducted in the absence of any commercial or financial relationships that could be construed as a potential conflict of interest.

## Publisher’s note

All claims expressed in this article are solely those of the authors and do not necessarily represent those of their affiliated organizations, or those of the publisher, the editors and the reviewers. Any product that may be evaluated in this article, or claim that may be made by its manufacturer, is not guaranteed or endorsed by the publisher.
