# Supplementary material for: Immunogenicity of two COVID-19 vaccines used in India: An observational cohort study in health care workers from a tertiary care hospital
Source: Front Immunol. 2022 Sep 23;13:928501. doi: 10.3389/fimmu.2022.928501 (PMC9540493; doi:10.3389/fimmu.2022.928501)
Supplement: Supplementary file 1 [file DataSheet_1.docx]

Supplementary Material

**Supplementary Table 1: Antibody responses in COVISHIELD recipients**

| **Parameter** | **Post-dose 2**  **(n=120)** |
| --- | --- |
| **Seroconversion (ELISA)** | 120 (100%) |
| **Seroconversion (PRNT)** | 114 (95%) |
| **PRNT titers** | |
| **Negative (<10)** | 6 (5%) |
| **10-<20** | 6 (5%) |
| **20-<50** | 36 (30%) |
| **50-<100** | 29 (24.1%) |
| **> 100** | 49 (40.8%) |

**Supplementary Table 2** Neutralizing antibody titres among COVISHIELD recipients grouped as pre-negatives and pre-positives at one month and 6months post-immunization

| **Variable** | **Pre-negatives (n=56)** | | | | **Pre-positives (n=42)** | | | |
| --- | --- | --- | --- | --- | --- | --- | --- | --- |
|  | **N** | **Median** | **IQR** | **p value*** | **N** | **Median** | **IQR** | **P value*** |
| **Overall** | 56 | 22 | 2.5-87 | <0.001 | 42 | 810 | 371-1297 | <0.001 |
|  | 120 | 64.4 | 34.5-154.2 |  | 67 | 1740 | 911-3116 |  |
| **Age<55yr** | 47 | 24 | 2.5-205 | 0.001 | 41 | 803 | 365-1316 | <0.001 |
|  | 95 | 70 | 36-149 |  | 64 | 1714.5 | 911-2732 |  |
| **Age>55yr** | 9 | 18 | 2.5-34 | 0.044 | 1 | 1050 | 1050-1050 | NA |
|  | 25 | 52 | 26-154 |  | 3 | 3831 | 2046-5267 |  |
| **Males** | 22 | 25 | 4-313 | 0.07 | 20 | 978 | 602-1353 | 0.0036 |
|  | 53 | 59 | 33-107 |  | 35 | 1947 | 1092-3626 |  |
| **Females** | 34 | 19.5 | 2.5-56 | 0.024 | 22 | 555 | 340-1150 | 0.004 |
|  | 67 | 69 | 35-155 |  | 32 | 1412 | 610-2337 |  |
| **Comorbidity (No)** | 39 | 27 | 12-383 | 0.026 | 31 | 802 | 377-1285 | 0.003 |
|  | 74 | 85 | 39-213 |  | 52 | 1957 | 899-8694 |  |
| **Comorbidity (yes)** | 14 | 16.5 | 2.5-192 | 0.11 | 6 | 902 | 604-1087 | 0.02 |
|  | 24 | 37 | 25-70 |  | 10 | 1502 | 1218-2094 |  |
| **BMI<25** | 27 | 26 | 14-341 | 0.16 | 16 | 700 | 357-1174 | 0.016 |
|  | 50 | 59 | 36-118 |  | 30 | 1714 | 659-3003 |  |
| **BMI>25** | 26 | 25 | 2.5-205 | 0.024 | 21 | 898 | 413-1316 | 0.0004 |
|  | 48 | 83 | 33-233 |  | 32 | 2005 | 1145-2560 |  |

**Supplementary Table 3: ELISPOT reactivity in relation to PRNT titers and prior exposure to SARS-CoV-2 post one month of vaccination (COVISHIELD)**

|  | **Reactive in ELISPOT/ total No tested (Individual SFU/million PBMCs values for the reactives)** | **Reactive in ELISPOT/ total No tested (Individual SFU/million PBMCs values for the reactives** |
| --- | --- | --- |
| **PRNT50 titer** | **Vaccinees without prior exposure** | **Vaccinees with   prior exposure** |
| **Negative** | 4/6 (303, 675, 480, 192) | None* |
| **10-<20** | 2/4 (21, 61) | None* |
| **21-50** | 5/10 (41,33, 220,22, 25) | None* |
| **51-100** | 4/9 (20, 41, 33, 141) | 1/2 (69) |
| **101-500** | 5/8 (429,20,1488,673,2149) | 2/2 (14,23) |
| **501-4000** | 6/8 (146,450,88,1784,52,99) | 3/9 (633,165,391) |
| **>4000** | None* | 1/5 (33) |
| **Total** | 26/45,57.8% (Median 21.7; IQR-1.6-169.2) | 7/18, 38.9% (Median 7.8; 0-41.9) |

*Among pre-positives, none of the participants exhibited PRNT titers <50 and among pre-negatives, titers > 4000 were not recorded.

The table denotes IFN-γ response (ELISPOT) in relation to PRNT titres and prior SARS CoV-2 exposure. For the responders identified by ELISPOT, values for SFU/million PBMCs are provided in the parentheses.

**
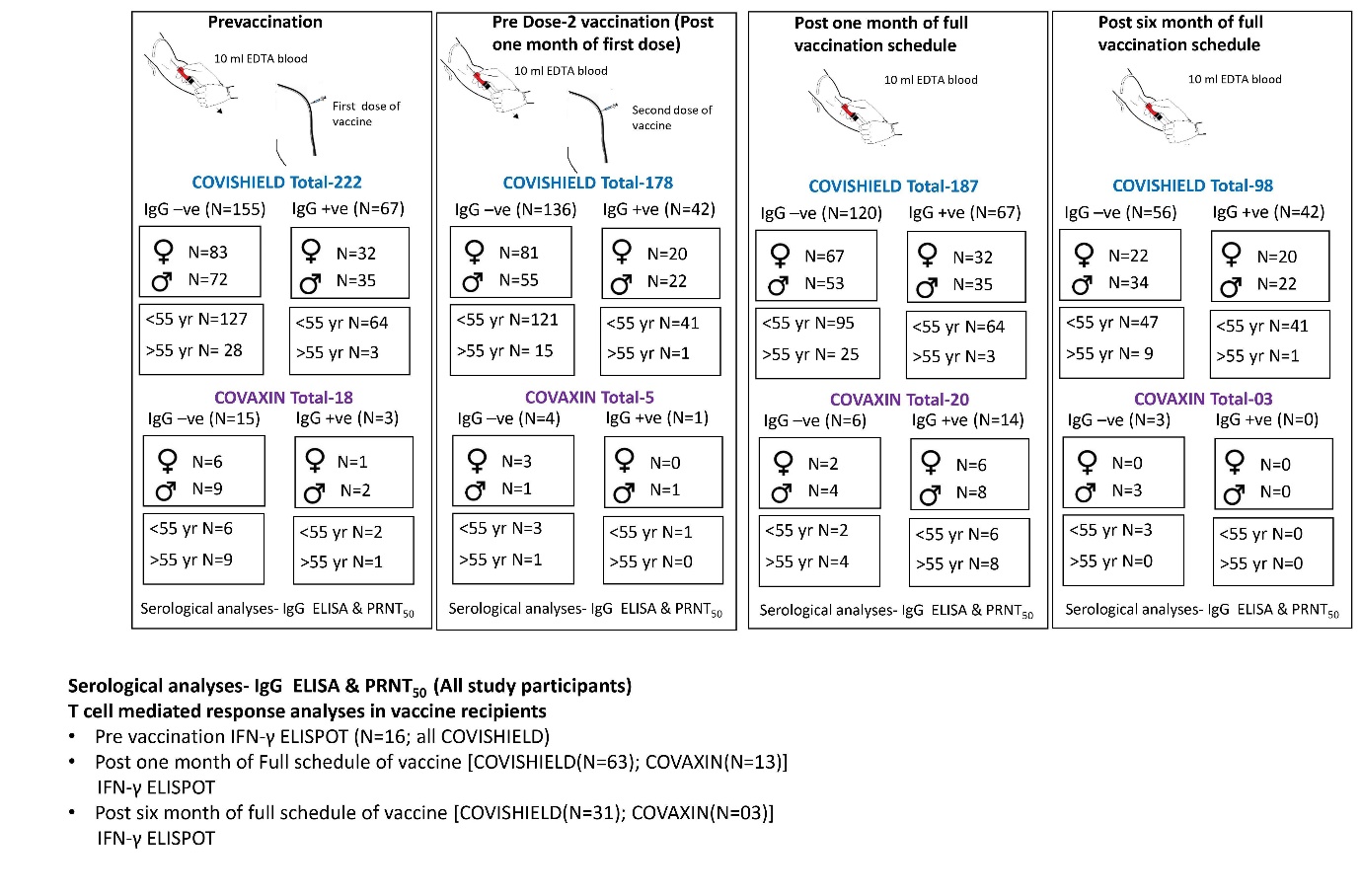
**

**Supplementary Figure-1: Study disposition.** The figure depicts age and gender wise categorization of the study participants based on SARS CoV-2 IgG positivity. The numbers studied at different time points i.e., pre-vaccination, Pre-dose 2, post 1month and post 6months -post 2^nd^ dose of both the vaccines are provided for each group.


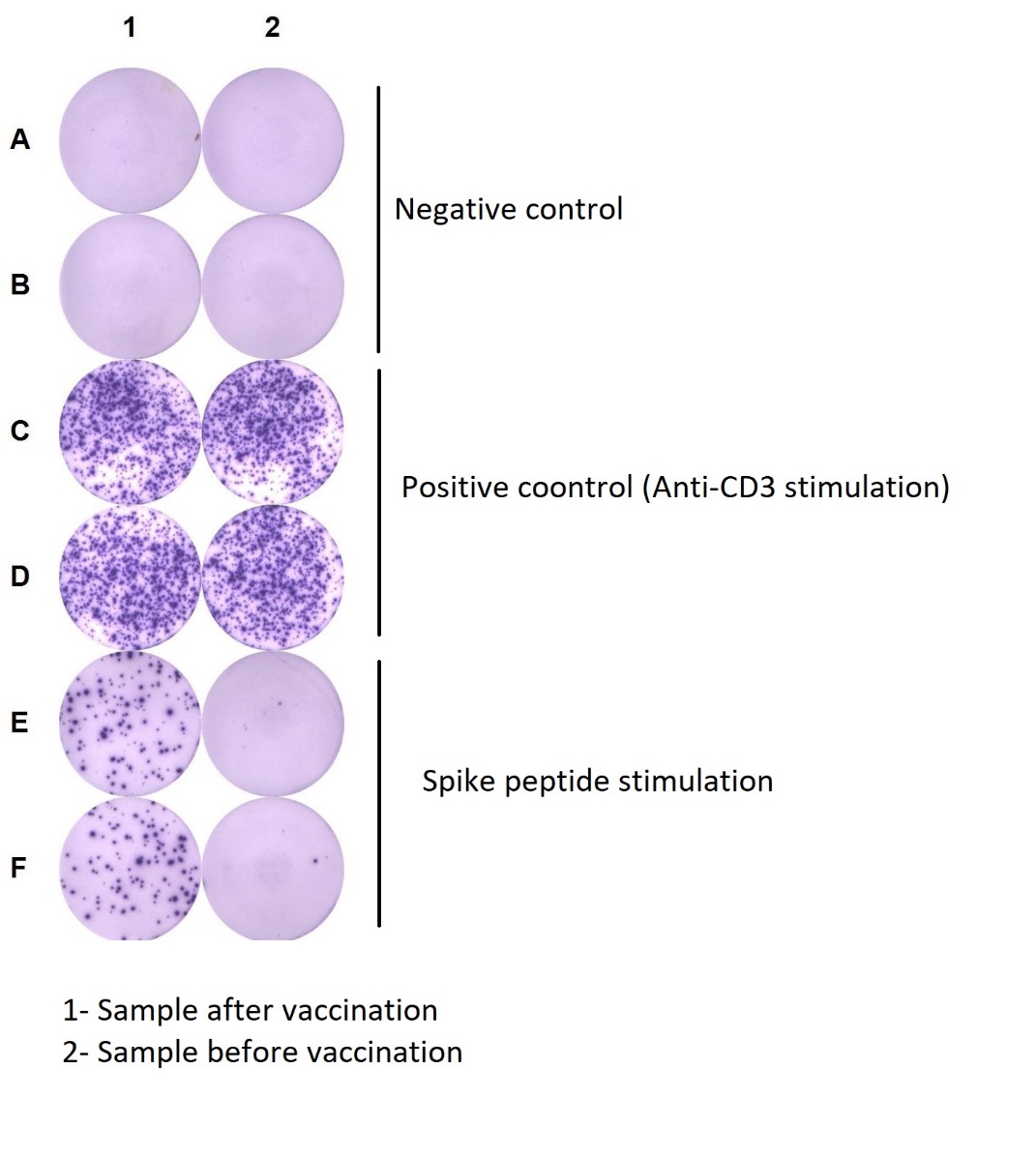


**Supplementary Figure-2: Representative image of IFN-γ ELISPOT assay performed for T cell immune response evaluation.** The figure shows the scanned image of IFN-γ ELISPOT strip plate comprising a single sample at baseline and after vaccination. The samples were stimulated with anti-CD3 antibody in positive control well whereas in test wells the spike peptides were used for stimulation. Only PBMCs with complete tissue culture media with 0.01% DMSO served as negative control.
